# Global inventory of species categorized by known underwater sonifery

**DOI:** 10.1038/s41597-023-02745-4

**Published:** 2023-12-18

**Authors:** Audrey Looby, Christine Erbe, Santiago Bravo, Kieran Cox, Hailey L. Davies, Lucia Di Iorio, Youenn Jézéquel, Francis Juanes, Charles W. Martin, T. Aran Mooney, Craig Radford, Laura K. Reynolds, Aaron N. Rice, Amalis Riera, Rodney Rountree, Brittnie Spriel, Jenni Stanley, Sarah Vela, Miles J. G. Parsons

**Affiliations:** 1 Fisheries and Aquatic Sciences, Institute of Food and Agricultural Sciences, University of Florida, Gainesville, FL USA; 2 Nature Coast Biological Station, Institute of Food and Agricultural Sciences, University of Florida, Cedar Key, FL USA; 3 Centre for Marine Science and Technology, Curtin University, Perth, WA Australia; 4 Instituto Oceanográfico, Universidade de São Paulo, São Paulo, SP Brasil; 5 Department of Biological Sciences, Simon Fraser University, Burnaby, BC Canada; 6 Department of Biology, University of Victoria, Victoria, BC Canada; 7 Centre de Formation et de Recherche sur les Environnements Méditerranéens, CNRS, Université de Perpignan Via Domitia, Perpignan, France; 8 Biology Department, Woods Hole Oceanographic Institution, Woods Hole, MA USA; 9 Stokes School of Marine and Environmental Sciences, University of South Alabama and Dauphin Island Sea Lab, Dauphin Island, AL USA; 10 Institute of Marine Science, Leigh Marine Laboratory, University of Auckland, Warkworth, New Zealand; 11 Soil, Water, and Ecosystem Sciences Department, Institute of Food and Agricultural Sciences, University of Florida, Gainesville, FL USA; 12 K. Lisa Yang Center for Conservation Bioacoustics, Cornell Lab of Ornithology, Cornell University, Ithaca, NY USA; 13 The Fish Listener, Waquoit, MA USA; 14 Coastal Marine Field Station, School of Science, University of Waikato, Tauranga, New Zealand; 15 MERIDIAN, Halifax, NS Canada; 16 Dalhousie University, Halifax, NS Canada; 17 Australian Institute of Marine Science, Perth, WA Australia

**Keywords:** Behavioural ecology, Marine biology, Freshwater ecology

## Abstract

A working group from the Global Library of Underwater Biological Sounds effort collaborated with the World Register of Marine Species (WoRMS) to create an inventory of species confirmed or expected to produce sound underwater. We used several existing inventories and additional literature searches to compile a dataset categorizing scientific knowledge of sonifery for 33,462 species and subspecies across marine mammals, other tetrapods, fishes, and invertebrates. We found 729 species documented as producing active and/or passive sounds under natural conditions, with another 21,911 species deemed likely to produce sounds based on evaluated taxonomic relationships. The dataset is available on both figshare and WoRMS where it can be regularly updated as new information becomes available. The data can also be integrated with other databases (e.g., SeaLifeBase, Global Biodiversity Information Facility) to advance future research on the distribution, evolution, ecology, management, and conservation of underwater soniferous species worldwide.

## Background & Summary

Numerous aquatic and semi-aquatic species, including amphibians, annelids, cephalopods, cetaceans, crustaceans, fishes, pinnipeds, reptiles, and sirenians, use sound underwater for information acquisition and dissemination^1–11^. Sounds produced intentionally for the purposes of communication or navigation (i.e., active sounds), and incidentally through other activities (i.e., passive sounds) may both serve numerous ecological functions^5,12,13^, such as for environmental sensing^14^, predator or prey detection^15–17^, and competition^18,19^. Additionally, the remote sensing of these sounds through passive acoustic monitoring (i.e., an observational method to detect and characterize sounds) has applications for invasive species detection^20,21^, habitat and species management^22,23^, ecological monitoring^24^, and optimization of aquaculture operations^25^, among other possible uses. Underwater sounds and their uses nonetheless face increasing threats from human activities and resulting environmental degradation^26–30^.

Despite decades of research, documentations of underwater sonifery across different taxa remain highly variable. For example, the majority of whales, dolphins, and porpoises have been confirmed to produce sound^31^. In contrast, fishes and underwater invertebrates have been relatively less studied when considering the thousands of extant species^5,32,33^. There have been several recent efforts to review sound production information for larger taxonomic groups globally (e.g., marine mammals^31^, marine invertebrates^33^, fishes^34^), but these have varied in their approaches and definitions of sonifery. As such, a comprehensive inventory of all known underwater soniferous species remains lacking, limiting the ability to document, summarize, or quantify trends in global underwater sound production^35^.

To address previous data limitations, the effort to create a Global Library of Underwater Biological Sounds (GLUBS)^35^ in collaboration with the World Register of Marine Species (WoRMS) created a dataset of information on likely or documented underwater sonifery in aquatic species. The World Register of Marine Species (available at MarineSpecies.org) provides a comprehensive, searchable database of known marine organisms and other taxa, featuring taxonomic information—including numerous accepted, synonymized, misspelled, and unaccepted classifications—along with a growing list of ecological attributes and traits^36–38^. Through its own datasets and connections or associations with other global datasets (e.g., the Ocean Biogeographic Information System, OBIS), the data presented on WoRMS can be used to answer a variety of questions related to distribution, evolution, ecology, management, conservation, and other fields worldwide^36^. The WoRMS database is widely used among researchers, with consistent growth in visitors since its inception in 2007, and at least 7,000 publications that have cited or mentioned WoRMS and its related registers^37,39^. As part of ongoing initiatives related to the United Nations Decade of Ocean Science for Sustainable Development (2021–2030) and Above and Beyond - Completing the World Register of Marine Species (ABC WoRMS), WoRMS is encouraging the documentation of species traits to better aid ecological research and provide more comprehensive information to its diverse user base^40^. Trait information on underwater sonifery by aquatic and semi-aquatic species is in service of these goals.

**Fig. 1:**
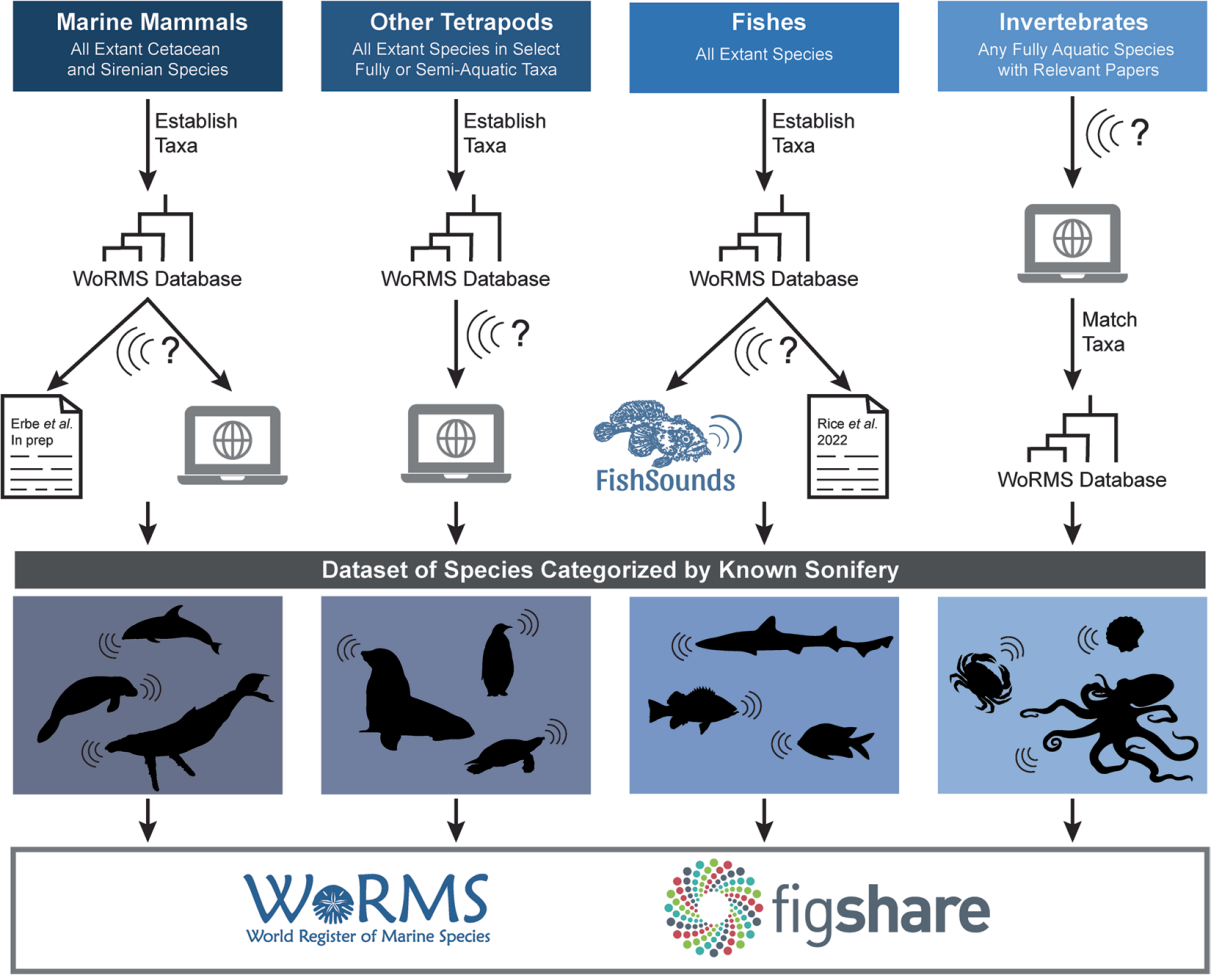
Conceptual diagram of the data collection methods used to create a dataset of species categorized by known sonifery. For the marine mammals, other tetrapods, and fishes, species and subspecies in each taxa were established based on the World Register of Marine Species (WoRMS) database, which were subsequently categorized based on existing sources and additional online literature searching. For invertebrates, online literature searching identified species that had been studied for their sound production, which were then matched to their appropriate listing on the WoRMS database. The resulting dataset was then published on WoRMS and figshare.

**Fig. 2:**
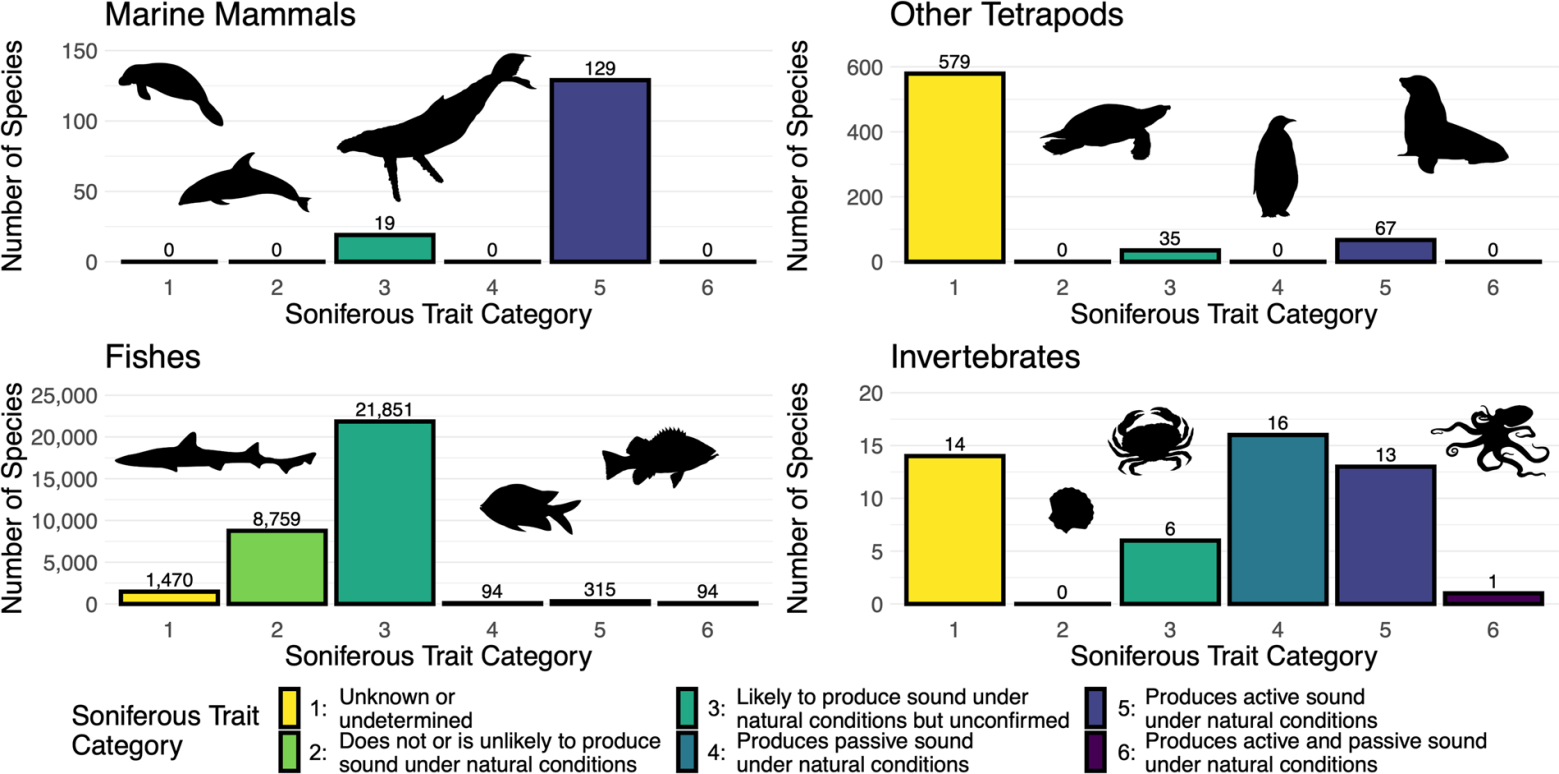
Bar graphs showing the number of aquatic and semi-aquatic species and subspecies placed in each soniferous trait category. The panels separate each taxa grouping, with images included to represent them.

Here, we present a global inventory of 33,462 aquatic and semi-aquatic species and subspecies categorized based on known underwater sound production. To create this dataset, we integrated existing sources for fishes, mysticetes, and odontocetes, and we conducted additional literature surveys for other semi-aquatic and aquatic taxa (Fig. 1). This trait has six categories according to whether a species has been confirmed or is considered likely to produce sound based on the available scientific literature (Table 1) following established definitions of active and passive sound production in natural and unnatural conditions, among other terms (Table 2). We found 729 species documented as producing active and/or passive sounds under natural conditions, with another 21,911 species deemed likely to produce sounds based on evaluated taxonomic relationships (Fig. 2). This dataset is available in the figshare data repository and WoRMS.

**Table 1:** Trait categories and their associated definitions used to describe soniferous behavior on the World Register of Marine Species.

Identifier	Presented information/field	Definition
Trait	Species exhibits underwater soniferous behavior	Does the species actively or passively produce sound under natural conditions while submerged in water?
Category 1	Unknown or undetermined	There is no known acoustic, morphological, or physiological study of sound production for this species and its likelihood of soniferous behavior based on an ancestral state reconstruction analysis, lineage, or evolutionary records has not been assessed, or this species has yet to be categorized by an expert source.
Category 2	Does not or is unlikely to produce sound under natural conditions	Species has been the subject of one or more studies to assess its acoustic behavior, morphology, and/or physiology, none of which have provided evidence of sound production either actively or passively, and/or species does not have a likelihood of soniferous behavior based on an ancestral state reconstruction analysis that results in a probability below 0.5, lineage, or evolutionary records.
Category 3	Likely to produce sound under natural conditions but unconfirmed	There is no known acoustic documentation to confidently validate sound production under natural conditions by this species, but it is likely to exhibit natural soniferous behavior based on an ancestral state reconstruction analysis that results in a probability of 0.5 or higher, lineage, evolutionary records, morphological characteristics, physiological characteristics, documented sound production behavior under artificial conditions, and/or documented sound production behavior with some uncertainty.
Category 4	Produces passive sound under natural conditions	There is validated acoustic documentation that this species produces passive sound under natural conditions, but no validated documentation to confirm that it actively produces sound.
Category 5	Produces active sound under natural conditions	There is validated acoustic documentation that this species produces active sound under natural conditions, but no validated documentation to confirm that it passively produces sound.
Category 6	Produces active and passive sound under natural conditions	There is validated acoustic documentation that this species produces active and passive sound under natural conditions.

**Table 2:** Terms and their associated definitions used to describe soniferous behavior on the World Register of Marine Species.

Term	Definition
Active sound	Produced deliberately in association with a particular behavior or situation, frequently with specialized sonic organs or structures, and generally used for communication; also referred to as intentional, deliberate, or specialized sounds.
Passive sound	May not be associated with specialized sound-producing structures nor with specific behaviors or situations, though may still serve some signal function; also referred to as incidental, unspecialized, or mechanical sounds.
Natural conditions	In the absence of artificial manipulation, such as an electrical current or direct handling.
Acoustic study or documentation	Documentation in the peer-reviewed or grey scientific literature of the presence or absence of audible sounds through listening live or on a recording in captivity or the field.
Morphological or physiological study or documentation	Documentation in the peer-reviewed or grey scientific literature of the presence or absence of morphological and/or physiological structures with sound production functions.
Ancestral state reconstruction analysis	The extrapolation back in time from measured characteristics of individuals to their common ancestors using statistical techniques to, in this case, recover information on the likelihood of soniferous behavior.
Artificial conditions	Involving human intervention or manipulation, such as an electrical current or direct handling.
Uncertainty	Doubt or uncertainty expressed by researchers conducting examinations for sound production about their conclusions (e.g., that the species they examined was correctly identified, that they correctly identified active versus passive sounds).
Validated	The researchers’ conclusions have no associated uncertainty in the documentation (see ‘Uncertainty’).

See Looby *et al*.^5^, Joy *et al*.^41^ and Rice *et al*.^53^ for additional information.

## Methods

### Trait and category definitions

The ecological trait, ‘species exhibits underwater soniferous behavior,’ and its respective categories were defined by a working group of seven scientists, all experts in bioacoustics and the research literature of a variety of aquatic taxa including mammals, fishes, and invertebrates. The various definitions were largely adapted from other recent works (e.g., ancestral state reconstruction^41^; active sounds^5^). We also considered whether sounds were produced naturally, based on long-standing concerns of artificial instigation creating unnatural or involuntary sound production behaviors^42–44^. We defined sounds produced under natural conditions as those made in the absence of artificial manipulation, such as an electrical current or direct handling, but may have included sounds made in captive environments, such as tanks. The resulting table of the trait, categories, and definitions were then reviewed and approved by the WoRMS Data Management Team. The trait and category definitions created are listed in Table 1 and additional definitions of terms are provided in Table 2.

### Data collection

Our data collection efforts centered on extant species and subspecies as listed on WoRMS. We separated taxa into four groupings for the purposes of our data collection. These groupings allowed us to better respond to the wide disparities in species richness, numbers of publications on sound production, and the existence of soniferous species reviews and inventories for the different taxa (Fig. 1).

#### Marine mammals

In this context, ‘marine mammals’ refers to fully aquatic marine mammal species and subspecies within the infraorder Cetacea or order Sirenia. All other mammal species for this dataset were examined as ‘other tetrapods.’ We used WoRMS^38^ as our taxonomic authority and queried the WoRMS database for a list of all accepted, extant marine mammal species and subspecies to base our data collection on, encompassing 148 species and subspecies.

Marine mammal ecological trait categorizations were largely based on work conducted for an upcoming Springer book on Marine Mammal Bioacoustics by Erbe and colleagues, which will provide an overview of sound production in marine mammals. There are many hundreds of publications on the active sounds produced by marine mammals, and their sounds have been summarized in several reviews on specific species groups or regions (e.g., Erbe *et al*.^45^). Therefore, it was not necessary to undertake a comprehensive literature search for every species. Rather, for each species and subspecies, we aimed to cite a recent review or summary of its sounds; a publication that documented a great variety of sounds; or one of the original or earliest publications of its sounds—in this order. As WoRMS somewhat differed from other marine mammal taxonomic authorities, such as the Society of Marine Mammalogy^46^, and the taxonomy associated with marine mammals is in ongoing flux, we also provided geographic areas associated with each species or subspecies based on the publications cited. We only focused on marine mammal active sound production for this review.

There were 19 species and subspecies—mostly offshore, cryptic species, such as some beaked whales (e.g., *Berardius minimus*)—whose sounds were not reported in available reviews or summaries. For these species, we searched Web of Science^47^ and Google Scholar^48^ for reports on their sound production in the peer-reviewed and grey literature. No records of their sounds were found. Based on the documented sounds for species within the same genera or families of these species, all these species with undocumented sound production were deemed ‘likely to produce sound under natural conditions, but unconfirmed’ (i.e., Category 3).

#### Other tetrapods

For the purposes of this dataset, ‘other tetrapods’ can be summarized as aquatic and semi-aquatic species and subspecies that occur in the following taxa groups: amphibians, hippopotamids, murids, mustelids, penguins, pinnipeds, ursids, and reptiles including crocodilians, lacertids, serpents, and testudines. For each of these groups of taxa, we queried the WoRMS^38^ database for a list of all accepted, extant marine species and subspecies. Though marine was specified, WoRMS defines ‘marine’ generously, so our taxa also included species that may predominantly live in and around brackish or fresh water or that may only be semi-aquatic (e.g., *Alligator mississippiensis*, *Hippopotamus amphibius, Rana aurora*).

Using Google Scholar^48^ and Scopus^49^, for each species and subspecies, we searched for papers that included “[scientific name]” OR “[common name]” AND “sound” OR “call” OR “acoustic” OR “vocal” OR “noise” in the title or keywords. We confirmed that papers within the search list referred to underwater sound production of the species in question and evaluated the content using the categories of sound production detailed in Table 1. For each species and subspecies, when applicable, the oldest reference that identified sound production under natural conditions was provided as the source of information for the WoRMS database. On occasion, a later source was retained, instead, if it had more information on the repertoire or behavior of the species than the oldest reference. Papers that provided information on an alternative or additional species outside of the list produced from WoRMS were retained for our dataset and added to the WoRMS database.

There has been no formal assessment (e.g., evolutionary analysis) of the likelihood of underwater sound production for these taxa. Additionally, for these taxa, few studies have been conducted where researchers have attempted to elicit underwater sound production. Therefore, for species that did not have a report of underwater sound production testing, the following actions were taken. If multiple species within the same family were found to produce sound, the species in question was deemed likely to produce sound under natural conditions. For example, most pinnipeds have reports of underwater sound production, but not all, and those that do not have associated reports have not been fully investigated for this behavior. As such, it was deemed likely that all pinniped species produce sound underwater, which can be confirmed when further investigations take place. Similarly, while few penguins reportedly exhibit this trait, the ones that have been studied have produced active sound underwater. Thus, all penguin species without an associated report have currently been assigned to Category 3, as well. For taxa where studies and reports of sound production are few (e.g., crocodilians, mustelids, rodents), species that did not have an associated report of sound production were assigned to Category 1 (i.e., unknown or unconfirmed).

#### Fishes

For the purposes of this dataset, fishes were defined as any extant, accepted species (not only marine) in the Subphylum Vertebrata, except for any species in the Megaclass Tetrapoda, as listed on WoRMS^38^. This includes the taxa Agnatha, Chondrichthyes, Sarcopterygii, and Actinopterygii. A complete list of fish species for use in the data collection was downloaded from WoRMS using either the WoRMS website or the R packages taxize^50^ and worms^51^.

The categorizations for fishes were adapted from two existing data sources: FishSounds^5,34,52^ and Rice *et al*.^53^. Both data sources used different criteria from the sonifery categories defined for WoRMS to determine the likelihood of sonifery in fishes. Nonetheless, they represent comprehensive resources of scientific knowledge about soniferous fish diversity and therefore allowed us to circumvent the need to re-review the full scope of literature on sound production in fishes, which comprise roughly 35,000 extant species^54^ and over 1,000 species that have been studied for sound production across more than 800 publications^55^.

The FishSounds website (available at FishSounds.net) presents a global inventory of fish species examined for sound production in the scientific literature. For each species studied in each reference, FishSounds reports whether the species has been found to produce sound, the sound production type as active and/or passive (as defined in Table 2), the varying methodologies used to examine the species for sound production, and the possibility for uncertainty (see Looby *et al*.^5^ for a complete description of the methods). The FishSounds dataset is therefore different in its sonifery determinations than the WoRMS categorizations described herein, but it provided an effective starting point for our data collection. The FishSounds dataset was retrieved from the FishSounds permanent data repository^52^. As the FishSounds dataset uses FishBase as its taxonomic source^54,56^, the species in the FishSounds dataset were matched to their respective accepted species entries on WoRMS using its Match Taxa tool^38^.

Using the FishSounds dataset, species were re-assigned to different WoRMS categories either automatedly or following a re-review process. If a species had been examined morphophysiologically or auditorily for sound production as defined by Looby *et al*.^5^ and found to be soniferous but with some amount of doubt in the authors’ conclusions or without visual confirmation of an auditory examination, then the species was placed in Category 3 and the FishSounds dataset was cited. To place fishes into Categories 4, 5, and 6 (Table 1), the FishSounds dataset was filtered to produce a list of species and their associated references that had examined the fish species for sound production auditorily with visual confirmation and had found the species to produce active and/or passive sounds, without doubt or uncertainty associated with their species identification, whether sounds were produced, nor the type of sound produced—all as determined following Looby *et al*.^5^ The references in the resulting list were then reviewed to determine whether the fish species produced the reported sounds under natural conditions. A total of 819 species studied across 613 references were assessed this way.

For the species that needed to be re-reviewed, if sound production was reported as naturally occurring in at least one reference in the FishSounds dataset, those species were placed in the appropriate Category 4, 5, or 6, depending on the types of sounds produced as reported in the references. If not, they were placed in Category 3. Because of the widespread use of direct manipulation to elicit fish sound production in the scientific literature across time and taxa^42–44,57–62^, authors within each reference had to explicitly specify that sound production occurred without direct human manipulation to be considered natural. One or two references that documented sound production were provided for each of the species that were reviewed with the methods described in this and the previous paragraph. Not all references that studied a particular fish species for sound production were reviewed in this data collection effort, as in some cases a single reference was sufficient to determine a species belonged in Category 4, 5, or 6. Additionally, the references cited for each category are not necessarily the oldest or most comprehensive descriptors of sound production behaviors—they may have just been the first reviewed that reported natural sound production. We recommend viewing the original FishSounds dataset for a more detailed overview of the sound production studies conducted for each species^52^.

Rice *et al*.^53^ conducted an ancestral state reconstruction analysis of sound production in Actinopterygii based on family-level sonifery data taken from the literature. Determinations made by Rice *et al*.^53^ of ancestral-state sonifery at the family level were extracted from their Supplemental Figure S1. The families were then used to determine whether species were likely to be soniferous (Category 3) or not likely to be soniferous (Category 2) based on these taxonomic relationships, for any species not covered by the FishSounds dataset. Such categorizations cited the Rice *et al*.^53^ publication as their determining source.

Species that were not included in the Rice *et al*.^53^ dataset and were examined but not found to produce sound in the FishSounds dataset were placed in Category 2 and the FishSounds dataset was cited. While it is generally assumed that all fish species are capable of making passive sound under certain conditions^13^, we did not include such an assumption in our categorizations as it remains unclear whether all fishes are likely to produce passive sounds naturally. Species that were not included in either dataset were placed in Category 1 and the FishSounds dataset was cited. Any fish species not listed in the provided dataset should be considered Category 1, as they were either absent from WoRMS, not listed as accepted species on WoRMS at the time of the review, or not covered under the definition of a fish species listed above.

#### Invertebrates

Since invertebrate species are so numerous with few that have been studied for their sound production, we only included species in our dataset for which we could find relevant publications. Any other species not listed in the dataset should be considered in Category 1 (unknown or undetermined) until further data can be collected or additional research is done.

We conducted both targeted and haphazard searches to compile data on invertebrate underwater sound production. We focused on several taxonomic groups that are more well-studied for their sound production. For crustaceans, we used the Web of Science Core Collection database^47^ and searched using the string: “TS = (crustacean*) AND ((TS = ((sound* OR noise* OR *acoustic* OR vocal*) NEAR/2 (mak* OR made OR produc* OR emit* OR communicat* OR call*))) OR (TS = (soniferous)))” following Looby *et al*.^5^ For mollusks, we searched by each class and group within the phylum (e.g., cephalopods) in Google Scholar using “[class name]” OR “[group name]” AND “sound” OR “call” OR “acoustic” OR “vocal” OR “soniferous”. We also searched haphazardly in Web of Science^47^, Google Scholar^48^, and personal reference libraries for any other reports of underwater sound production in these or other invertebrate taxa. Each publication found was read to determine its applicability to the data collection. The species studied were matched to their associated listing on WoRMS^38^ and placed in their appropriate categories accordingly.

## Data Records

The known sonifery data are available in the figshare data repository in a single Excel spreadsheet file^63^. The spreadsheet contains three tabs: a data dictionary, the reference information, and the sonifery information. The data dictionary provides information defining each column variable present in the other two spreadsheet tabs, including the variable name, allowed values, variable definition, and any needed additional information. The reference information provides the citation information (e.g., authors, publication name) for all the references used to categorize species by known underwater sonifery. The DOIs of the associated references were also provided whenever available to aid in locating the references. The sonifery information contains the taxon names, the soniferous categories assigned to them, and any notes that were included about the categorizations. To relate which reference was cited for each soniferous trait record, the reference information can be linked to the sonifery information through the provided Reference Aphia IDs. The reference IDs were kept consistent with the Aphia ID system utilized by WoRMS but can also be considered independently from WoRMS if using this dataset as a stand-alone source. As scientific names may change with future taxonomic reclassifications, to improve dataset sustainability, for each taxon, we also provided the Taxon Aphia ID as listed in WoRMS, though this may be ancillary or unnecessary to the dataset’s use. The dataset as provided on figshare has also been integrated into WoRMS, where it can be more readily updated with new soniferous information or taxonomic reclassifications. Any R code and associated data files used to generate or summarize the dataset are also provided in figshare^63^.

## Technical Validation

Data were collected by experts in their respective soniferous taxa using either existing data sources or through novel searches of the existing literature using systematized review methodology when possible^64^. The data collected were verified against other similar reviews^65^ or estimations of known soniferous species numbers^66–68^ when available to ensure the results provided herein were reasonable. Most of the data collected for the different animal groups identified in the methods were categorized by a single reviewer each, however, with limited external validation at the time of publishing. This may have introduced omissions or mistakes in the dataset based on human error or biases associated with the review methods. The WoRMS database allows for continual editing and so the authors, as WoRMS thematic experts, will continue to update and validate the data as new information becomes available or any corrections are needed. Any users of the compiled dataset should be aware of the possibility of errors and ongoing changes. Users are encouraged to verify any specific reports of sonifery using the references provided and to contact the authors of this data publication if any discrepancies are found.

As both active and passive sounds have potential ecological signaling and monitoring applications^5,12–25^, the dataset attempted to capture both types of sonifery in its categories. The definitions provided for these sound types in Table 2 were adapted from previously published text and commonly used delineations in the scientific literature that the dataset was meant to encompass^5,12,13,31,44^^,^. The distinction between passive and active sounds, however, may exist more along a spectrum as opposed to in stark contrasts^5^. For example, active sound production may be the result of exaptation in taxa where it is not an ancestral trait^69^, and passive sounds may be associated with particular behaviors or situations^5,70^. Determining the use of sounds for intentional communication can often be uncertain and may require further behavioral testing to designate conclusively^5,71–73^. The dataset’s categorizations also relied on reviewers’ interpretations of sound production descriptions in the surveyed references. This process may have simplified more nuanced sonifery descriptions in the source materials. Users are therefore highly encouraged to refer to the provided data sources for additional information on the context surrounding the documented sound production. Because likely many species are capable of passive sound production in certain contexts (e.g., through swimming), species were only categorized as passively soniferous in the dataset if their sounds were detectable in a natural context within a scientific study. We also did not include reports of passive sounds for the tetrapods in our data collection, though there are likely many species that produce them^31,71^.

Users should be aware of potential limitations or qualifications associated with our categorizations. While our trait and category definitions relied on widely used and established differentiations in the sound production literature, they were relatively conservative in their inclusion of species in Categories 4, 5, and 6 and may therefore have led to the exclusion of species that are indeed naturally soniferous. For example, for the purposes of our data collection, we emphasized species that were shown to produce sounds underwater naturally without contact, electricity, tethering, or other forms of direct manipulation by humans, while still including sounds spontaneously produced in artificial environments (e.g., tanks). This may have led to the exclusion of species that do indeed produce sounds naturally, such as species who may make distress sounds in response to being caught by predators or those that readily produce sounds when handled but may have simply not yet been shown to do so in other agonistic situations^7,43,59,74–76^. Due to frequent uncertainty in studies of underwater sound production^5^, species may have been similarly excluded from Categories 4, 5, or 6 if their potential sonifery could not be confirmed in the surveyed studies. Additionally, for some taxa, we relied on ancestral state reconstruction analyses or assumptions of sonifery based on taxonomic relationships to determine species likely to be soniferous. There is, nonetheless, the possibility for sound production behaviors to be secondarily lost, thus the species deemed likely soniferous should be treated with caution^53,77–80^. Users should be aware of the assumptions and simplifications made by our categorizations, the potential for our understanding of sound production and likely sound production to change, and that the data may later be updated to reflect new research even if the categories themselves may not change.

## Usage Notes

We envision numerous possible applications for the dataset described herein to study sound production and other topics related to aquatic and semi-aquatic species worldwide. By listing a soniferous behavior trait on WoRMS, researchers will be able to easily search for and access known sonifery information for any species encompassed by our review. This will aid in identifying both known soniferous species and species lacking documented sound production. In addition to being listed on WoRMS, the soniferous trait data associated with this review is available in a spreadsheet file in figshare that may be easier to use in analyses. The ecological trait data can be easily integrated with other information available on WoRMS itself (e.g., taxonomy, environment) or its associated databases (e.g., conservation status, distribution, invasive status) to study global patterns of sound production behavior and the use of passive acoustics for ecological monitoring. Sonifery has been studied in these contexts before, but only with more limited datasets^5,53,65^. Researchers interested in conducting such analyses should be aware of the availability of software packages that could be used to easily access related datasets (e.g., the rfishbase^56^, taxize^50^, worms^51^, and rinat^81^ packages in R). We hope our data will facilitate future research on the distribution, evolution, ecology, management, and conservation of underwater soniferous species worldwide.

## Data Availability

Code and associated data files are available in the figshare data repository^63^.
